# Attitudes toward sexual and reproductive health and rights and their associations with reproductive agency: a population-based cross-sectional study in Ethiopia, Kenya, and Zimbabwe

**DOI:** 10.1080/26410397.2024.2444725

**Published:** 2025-01-13

**Authors:** Karin Båge, Anna Kågesten, Olalekan Uthman, Mariano Salazar, Bi Puranen, Signe Svallfors, Anna Mia Ekström, Helena Litorp

**Affiliations:** a Phd Student, Department of Global Public Health, Karolinska Institutet, Stockholm, Sweden.; b Associate Professor, Department of Global Public Health, Karolinska Institutet, Stockholm, Sweden; c Professor, Warwick Centre for Global Health, Warwick Applied Health, Warwick Medical School, Warwick University, Warwick, UK; d Associate Professor, Department of Global Public Health, Karolinska Institutet, Stockholm, Sweden; e Associate Professor, Senior Research Fellow, Institute for Future Studies, Stockholm, Sweden; Secretary General, World Values Survey, Stockholm, Sweden; f Post Doctoral Fellow, Department of Sociology, Stanford University, Stanford, CA, USA; Post Doctoral Fellow, Department of Sociology, Stockholm University, Stockholm, Sweden; Post Doctoral Fellow, Department of Global Public Health, Karolinska Institutet, Stockholm, Sweden; g Professor, Department of Global Public Health, Karolinska Institutet, Stockholm, Sweden; Senior Physician, Department of Infectious Diseases/Venhälsan, South General Hospital, Stockholm, Sweden; h Associate Professor, Department of Global Public Health, Karolinska Institutet, Stockholm, Sweden; Associate Professor, Department of Women’s and Children’s Health, Uppsala University, Uppsala, Sweden

**Keywords:** sexual and reproductive health and rights, attitudes, reproductive agency, reproductive empowerment, Ethiopia, Kenya, Zimbabwe, World Values Survey

## Abstract

We investigated the association between values and attitudes toward sexual and reproductive health and rights (SRHR) and gender equality, with reproductive agency in Ethiopia, Kenya, and Zimbabwe. Using 2020-21 World Values Survey (WVS) data (*n* = 3096), we utilised the SRHR Support Index including five subindices to gauge SRHR attitudes, the WVS Equality Index for gender equality values, and the perceived level of freedom of choice and control over whether, when, and how many children to have as a proxy for reproductive agency. Descriptive statistics, bivariate, and multivariable logistic regressions were used to analyse how values and attitudes differed between respondents of high vs low reproductive agency using the median as cut-off, stratified by country and sex. Country, education, subjective social class, and religion were associated with reproductive agency. Adjusted analyses indicated associations between supportive values and attitudes towards equitable masculinity norms, SRHR interventions and gender equality, with high reproductive agency. Associations varied more between countries than by sex. Findings suggest an association between SRHR and gender equality values and attitudes and the level of reproductive agency, and underscore the importance of addressing values and attitudes in context-specific interventions. Measures of SRHR progress should be critically reviewed and complemented with self-assessed – as opposed to researcher-ascribed – items to support the successful implementation of global SRHR agendas.

## Plain language summary

People’s reproductive health depends on their freedom of choice and control over childbearing. This is what the concept “reproductive agency” means. In this study, we explored people's values and attitudes toward sexual and reproductive health and rights (SRHR) and towards gender equality. We then analysed whether these values and attitudes were linked to their reproductive agency. We used data from the 2020-21 World Values Survey (WVS) in Ethiopia, Kenya, and Zimbabwe. The data included 3,096 respondents. We measured values and attitudes using two indices: the SRHR Support Index and the WVS Equality Index. To measure reproductive agency, we asked people if they thought they were free to choose and control if, when, and how many children to have. We categorised the responses into two groups: high and low reproductive agency. We used statistical methods to compare values and attitudes between these two groups in the three countries. We also compared values and attitudes between men and women. Reproductive agency varied by country, education, social class, and religion. People who were supportive of SRHR and gender equality were slightly more likely to report high reproductive agency. We saw this particularly for those who were supportive of equitable masculinity norms, SRHR interventions (like safe abortion), and gender equality. The links varied more between countries than between the sexes. To improve SRHR for all, it is essential to address values and attitudes and underlying norms in ways that are relevant to the context. Self-assessed questions could be added to surveys to better align measures of progress with the goals of global SRHR agendas.

## Résumé

Nous avons étudié l’association entre les valeurs et les attitudes à l’égard de la santé et des droits sexuels et reproductifs et de l’égalité de genre, avec la capacité d’action en matière de procréation en Éthiopie, au Kenya et au Zimbabwe. À l’aide des données de l’Enquête mondiale sur les valeurs (WVS) 2020-2021 (*n* = 3096), nous avons utilisé l’indice de soutien à la santé et aux droits sexuels et reproductifs comprenant cinq sous-indices pour évaluer les attitudes en matière de santé et de droits sexuels et reproductifs, l’indice d’égalité de la WVS pour les valeurs d’égalité des sexes et le niveau perçu de liberté de choix et de contrôle sur le fait d’avoir des enfants, le moment et le nombre des naissances comme indicateur de la capacité d’action en matière de procréation. Des statistiques descriptives, des régressions logistiques bivariées et multivariées ont été utilisées pour analyser la manière dont les valeurs et les attitudes différaient entre les répondants ayant une capacité d’action reproductive élevée et faible, au moyen de la médiane comme seuil, stratifiée par pays et par sexe. Le pays, l’éducation, la classe sociale subjective et la religion étaient associés à la capacité d’action en matière de procréation. Les analyses ajustées ont indiqué des associations entre les valeurs et les attitudes de soutien à l’égard des normes de masculinité équitables, les interventions de santé et de droits sexuels et reproductifs et l’égalité des sexes, avec une capacité d’action reproductive élevée. Les associations variaient davantage entre les pays que selon le sexe. Les résultats suggèrent une association entre les valeurs et attitudes en matière de santé et droits sexuels et reproductifs et d’égalité des sexes et le niveau de capacité d’action en matière de procréation, et soulignent combien il est important d’aborder les valeurs et attitudes dans des interventions spécifiques au contexte. Les mesures des progrès en matière de santé et de droits sexuels et reproductifs doivent être examinées de manière critique et complétées par des éléments d’auto-évaluation – par opposition à des éléments attribués par les chercheurs – pour soutenir une mise en œuvre réussie des programmes mondiaux de santé et de droits sexuels et reproductifs.

## Resumen

Investigamos la asociación entre valores y actitudes relacionados con la salud y los derechos sexuales y reproductivos (SDSR) y la igualdad de género con la agencia reproductiva en Etiopía, Kenia y Zimbabue. Utilizando datos de la Encuesta Mundial de Valores (EMV) de 2020-21 (*n* = 3096), utilizamos el Índice de Apoyo para SDSR, incluidos cinco subíndices para medir las actitudes sobre SDSR, el Índice de Igualdad de la EMV para los valores de igualdad de género y el nivel percibido de libertad de elección y control de si tener o no hijos, cuándo tenerlos y cuántos tener como un indicador de la agencia reproductiva. Se utilizaron estadísticas descriptivas y regresiones logísticas bivariadas y multivariables para analizar las diferencias entre los valores y las actitudes de las encuestadas con mucha agencia reproductiva y aquéllas con poca agencia reproductiva, utilizando la mediana como punto de corte, estratificadas por país y sexo. El país, el nivel de escolaridad, la clase social subjetiva y la religión de las participantes estaban asociados con su agencia reproductiva. Los análisis ajustados indicaron asociaciones entre los valores y las actitudes que apoyaban las normas de masculinidad equitativas, las intervenciones de SDSR y la igualdad de género, con mucha agencia reproductiva. Las asociaciones variaron más entre países que por sexo. Los hallazgos indican una asociación entre los valores y las actitudes relacionados con SDSR e igualdad de género y el nivel de agencia reproductiva, y subrayan la importancia de abordar los valores y las actitudes en intervenciones realizadas en contextos específicos. Las medidas de los avances en SDSR deben revisarse de manera crítica y suplementarse con elementos autoevaluados, y no adscritos por investigadores, para respaldar la ejecución exitosa de las agendas mundiales de SDSR.

## Introduction

Sub-Saharan Africa (SSA) has achieved much progress in recent decades in improving sexual and reproductive health and rights (SRHR), despite struggling with some of the world’s worst SRHR outcomes.^1^ Owing to ambitious policy frameworks such as the Maputo Protocol,^2^ art.2.1 (a), SSA has seen significant reductions in metrics; maternal mortality declined by a third between 2000 and 2020,^3^ the prevalence of female genital mutilation/cutting (FGM/C) reduced to 8% from 71% in the East Africa region between 1995 and 2016,^4,5^ and HIV infections reduced by 56% in the last 14 years.^6^ Women’s and girls’ empowerment is recognised as a critical factor in accelerating this progress toward gender equality and sustainable development for all, as stipulated by the 17 Sustainable Development Goals (SDGs) of Agenda 2030.^7^

According to the new, comprehensive definition of SRHR launched by the Guttmacher-Lancet Commission in 2018, achieving sexual and reproductive health (SRH) for all is conditional on the realisation of people’s rights to make autonomous choices related to their own body and relationships free from violence or coercion, irrespective of the social, cultural, legal and economic context.^8^ SDG 5 on Gender Equality tracks women and girls’ ability to say no to sex, and freely decide on the use of contraception and their healthcare.^9^ In SSA, less than half (48%) of women and girls make independent decisions regarding all three dimensions and are considered fully empowered.^10^ Despite the principle of universality of SRHR, contextual factors do matter for individuals’ ability to fulfil their SRHR.^8,10,11^

### Empowerment and agency

To theoretically clarify the relationships between individuals, their contexts, and SRHR outcomes, we use a comprehensive framework for reproductive empowerment developed by the International Centre for Research on Women (ICRW).^12^ Reproductive empowerment is understood as a multifaceted concept that is “both a process and an outcome”.^12^Central to this framework is “reproductive agency” which refers to people’s capacity to take meaningful action to fulfil their reproductive aspirations and choices.^12^ The framework is based on a social-ecological model and emphasises relational aspects; reproductive empowerment and agency are dynamic, non-linear, and can change both over time and between different settings.^12^ While agency and empowerment are interrelated, we focus on the term “agency” in this paper but refer to both terms to reflect the literature.

Recognised as a multidimensional concept that can be complex to measure, women’s empowerment is often understood as a key pathway to achieving gender equality through its emphasis on women’s agency, autonomy, control, and choice.^12–14^ In the SRHR literature, women’s empowerment has been measured in a variety of ways, including but not limited to socioeconomic resource levels such as education, media engagement, and access to income; decision-making power over health care, household income, and expenses; as well as reproductive behaviour and attitudes to gender-based violence (GBV).^15–21^ These operationalisations of women’s empowerment have mostly been studied in relation to SRH outcomes for women, such as fertility rates, unmet need for contraceptive use, exposure to GBV, and other maternal and child health outcomes.^21–26^ In quantitative surveys, people are rarely asked how they perceive their level of empowerment, agency, or autonomy, regardless of how this is defined, nor how it is influenced by individual and social factors including sociodemographic characteristics as well as values and attitudes toward SRHR and gender equality.^26^

Studies that specifically aim to develop scales to measure reproductive agency or related concepts, mostly rely on pre-determined notions.^27–30^ For example, assessments of agency are based on how women answer questions related to knowledge of contraceptive methods, behaviour during pregnancy, or decision-making power over family planning.^27–30^ The scales and indices that do integrate items that provide respondents with opportunities to subjectively assess their own situation, face challenges related to scale-up for global systematic data collection and analysis.^31,32^ Despite the (much-needed) comprehensive approach of studies that try to capture the multilayered aspects of agency, researcher-ascribed definitions of empowerment or agency tend to be used over person-centred items.^33,34^ Because of the lack of subjective assessments, there is an overemphasis in the literature on the instrumental value of empowerment or agency for achieving SRH, rather than as a right or subjective experience with intrinsic value.^12,35,36^

### Attitudes, values, norms, SRHR and gender

Individual-level values and attitudes have long been understood to influence health-related behaviour, including SRH. For example, research show how attitudes toward condoms predict their use among adolescents in Spain,^37^ that gender-equitable attitudes among male partners are associated with increased use of modern birth control among couples in Ethiopia and Kenya,^38^ and how gender-equitable attitudes are associated with less violent behaviour and higher sexual satisfaction among men.^39^ In empowerment research, individual-level attitudes toward various aspects of gender equality – primarily interpersonal violence or GBV – have been conceptualised as intrinsic aspects of agency.^20,40,41^

In this study, we argue that individual-level values and attitudes should be understood as determinants, rather than indicators of agency. We draw on research from psychology, where studies on value-based action indicate how the agency to act according to one’s values enhances psychological well-being,^42,43^ and structural sexism theory which explains how attitudes related to gender roles influence women’s health through impacting their agency.^44^ While values and attitudes can be thought of as different concepts, we use both terms in this study to refer to individually held beliefs, and to distinguish them from social norms. This also reflects the differing terminology used by the World Values Survey (WVS) and the SRHR/public health field.^45^

Values and attitudes are nested in normative contexts, particularly related to the social expectations regarding what is considered appropriate behaviour, roles, and responsibilities of men and women as well as the power relationships between and within them – defined as gender.^46^ While these gender constructs vary across contexts,^46–50^ they influence a person’s understanding of, and ability to exercise agency, ultimately contributing to sustaining gender inequalities and impeding universal fulfilment of SRHR.^12,51^ We argue that individual-level values and attitudes are relevant to explore. Not only are they understood as critical factors that inform behaviour, especially when these are not aligned with prevailing norms,^12^ but also global data indicate that women’s access to external empowerment opportunities such as education is facilitated by, and depends on, values and attitudes that support women’s empowerment.^52^ We therefore hypothesise that individuals’ values and attitudes toward SRHR and gender equality are linked to their perceived level of reproductive agency.

We also recognise that other factors can confound this association, such as a person’s gender and sex, education, and family size.^20,44,53,54^ In addition, marital status, religion, place of residency, country, and subjective social class may also influence one’s sense of (reproductive) empowerment or agency.^13,20,53,55,56^ Age and place of residence (urban or rural) may impact reproductive agency in different ways. For example, a young person may not feel as if they have freedom of choice and control over their reproductive life due to a lack of financial resources, access to contraceptives, knowledge, experience, and independence. An older person may equally rate their reproductive agency as low due to the biological decline in fertility. Likewise, while SRHR services may be more available in urban areas, rural communities may have a stronger support system and a sense of belonging, which may impact perceived reproductive agency.

### Study aim

The aim of this study is two-fold. First, we examine the extent to which individuals experience reproductive agency in three SSA countries: Ethiopia, Kenya, and Zimbabwe. Our nationally representative samples includes both adult men and women, regardless of marital status and place of residence. Second, we assess whether individuals’ values and attitudes toward SRHR and gender equality as well as their sociodemographic characteristics are associated with their perceived reproductive agency.

### Study countries

Ethiopia, Kenya, and Zimbabwe represent different African regions and health systems, with differing fertility rates, family planning services, maternal mortality ratio, levels of unmet need for contraception, and other SRHR metrics.^24,57^ According to UNDP’s Gender Inequality Index, Kenya and Zimbabwe are more gender-equal than Ethiopia.^58^ In Ethiopia, fewer than half of all women and girls (45%) can autonomously decide on contraceptive use, health care, and having sex, compared to Kenya and Zimbabwe where a majority of women and girls, 56% and 60% respectively, can do so.^10^

## Materials and methods

### Study design

This is a population-based, cross-sectional study using quantitative data on values collected among adult men and women in Ethiopia, Kenya, and Zimbabwe via the 7th round of the World Values Survey (WVS). The WVS is an international social research network that has collected quantitative data on values in 120 countries across the world since 1981.^59^ Data for our study was collected in Ethiopia and Zimbabwe in March–May 2020, and Kenya in August 2021. Each country has a local Principal Investigator and local interview teams carry out data collection, including reporting feedback. Interviews are carried out in local languages spoken by at least 15% of the population.^60^ Kantar Public Nigeria carried out all three surveys. Detailed data collection procedures can be found at the WVS website (www.worldvaluessurvey.org).

The sample size is based on the WVS nationally representative sampling design, generally aiming at about 1,200 men and women aged 18 or above per country.^61^ WVS Round 7 included a total of 3,666 participants from the three countries.

As described elsewhere,^45^ an SRHR and Gender module was developed in collaboration between the WVS and our team at Karolinska Institutet. Following piloting in Nigeria in 2017–2018,^56^ the module was slightly revised before being integrated into the WVS surveys in the three study countries. This module comprised 47 items across various dimensions of SRHR, covering topics such as early marriage, early childbearing, FMG/C, skilled attendant delivery, contraceptive use, and – critical to our study – perceived level of free choice and control over family planning. Parts of this module will be included in the WVS core questionnaire in the next wave.

### Dependent variable: low vs. high self-reported reproductive agency

We measured reproductive agency, as a dependent variable reflecting freedom of choice and control over one’s reproductive life. The survey question reads:

“Some men/women feel they have completely free choice and control over family planning (if, when and how many children to have), while others do not. Please use this scale where 1 means ‘no choice at all’ and 10 means ‘a great deal of choice’ to indicate how much freedom of choice and control you feel that you have over your family planning”.

As we rely on self-reported data, we interpreted this outcome variable as capturing subjective reproductive agency. Due to left-skew we dichotomised this indicator using the median of 9 as a cut-off point to examine whether respondents experienced low (1–8) or high (9–10) reproductive agency.^62^ Median (and IQR) values per country and sex were 9 (6–10) in Ethiopia; 8 (6–10) in Kenya; 10 (6–10) in Zimbabwe; 9 (6–10) among men; and 9 (6–10) among women.

### Independent variables

#### Values and attitudes related to SRHR

We relied on a newly developed SRHR Support Index based on the WVS SRHR and Gender module described above.^63^ This index comprises 23 Likert-scale items (Cronbach’s α = 0.80) divided into 5 sub-indices, comprehensive of several dimensions of SRHR as defined by the Guttmacher Lancet Commission:^8^ Sexual and Reproductive Rights Norms (Cronbach’s α = 0.92), Neighborhood Sexual Safety Norms (Cronbach’s α = 0.86), Gender-Equitable Relationships Norms (Cronbach’s α = 0.71), Equitable Masculinity Norms (Cronbach’s α = 0.65), SRHR Interventions Norms (Cronbach’s α = 0.57).^63^ For all items, respondents were asked to define the extent to which they agreed with statements related to SRHR (Box 1). The index and sub-indices were standardised on a scale of 0–100, where higher scores indicated more overall support for SRHR. As such, we treated the index and sub-indices as continuous variables.

#### Values and attitudes related to gender equality

To reflect the interconnections between gender equality and SRHR,^12^ we also included the pre-existing WVS index on values toward gender equality in the analysis. The Equality Index includes five items; (1) whether children suffer when a mother works for pay, (2) women’s capacity as political leaders and (3) as business leaders compared to men, (4) whether university education is more important for boys than girls, and (5) whether being a housewife is as fulfilling as paid employment. As this index is standardised on a scale of 0–1 where higher scores indicate higher support for gender equality, we recoded this variable to a scale of 0–100 to match the SRHR Support Index and its five subindices.

### Sociodemographic covariates

To capture the structural factors associated with reproductive agency as outlined by the conceptual framework, we also included various sociodemographic variables: country (Ethiopia, Kenya, Zimbabwe), sex (women, men), age (18–24, 25–29, 30–39, 40–49, 50–99), residency (urban, rural), level of education (low, middle, high), subjective social class (upper and upper middle class, working and lower-middle class, lower class)^1^, marital status (married/cohabiting, divorced/widowed/separated, single), religion (Christian, Muslim, no/other religion), and number of children (none, 1, 2–3, 4 or above).

### Statistical methods

After removing missing values on the included variables (Figure 1), we used descriptive statistics and Pearson’s chi-square tests to examine the distribution of participants’ reproductive agency and sociodemographic characteristics. This was followed by examining the distribution of the SRHR Support Index, its five sub-indices, and the WVS Equality Index disaggregated by level of reproductive agency, using mean, standard deviation, median, and interquartile range (IQR) summary statistics. We also visualised the distribution using histograms (Appendix Figures 1A and 2A).

**Figure 1.**
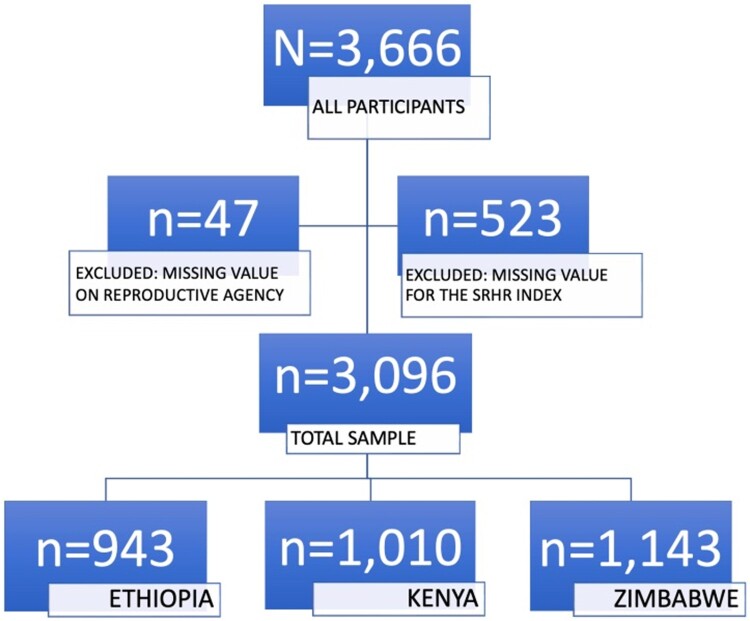
Flowchart showing inclusion and exclusion criteria for the study population

To test our hypothesis that individuals’ values and attitudes toward SRHR and gender equality are associated with their subjective reproductive agency, we estimated crude and adjusted odds ratios (cOR and aOR) with 95% confidence intervals (CI) using bi- and multivariate logistic regressions. These models measure the probability of experiencing high reproductive agency, as compared to low reproductive agency, using a logistic function. To control for possible confounding factors, we added the sociodemographic variables introduced above in the multivariate analyses. Results from the logistic regression were stratified by sex and country to detect potential differences between groups.

Akaike’s Information Criterion (AIC) and Bayesian Information Criterion (BIC) tests indicated that a logistic model fit the data better than linear regression. Since cross-model comparisons of estimates using logistic regressions can be imprecise,^64^ we replicated our analyses using linear probability models in sensitivity analyses (Appendix Table 2A). To account for local norms and other characteristics, we also ran a sensitivity check adding region-fixed effects (Appendix Table 3A). Data were analysed using STATA/SE 17.0.

### Reflexivity statement

Research involving humans, including quantitative studies such as our own, is always shaped by the social position of researchers.^65^ Our team comprises various gender identities, sexualities, ethnicities, geographic backgrounds, and levels of academic seniority. Together, we bring perspectives from social anthropology, public health, medicine, history, sociology, and demography. We have experiences of growing up, working, and living in different normative contexts, including two of the study countries of this paper. Further, the team members have extensive experience researching a range of SRHR dimensions (e.g. contraception and abortion, maternal health, HIV prevention and treatment, and gender norms, among adolescents and LGBTQIA+ people) in various contexts, including in the study countries and their wider region. As such, our team holds a diverse and interdisciplinary perspective on SRHR and gender norms.

### Ethical considerations

The WVS Scientific Advisory Board and the WVS Archive control data collection procedures to ensure data quality and confidentiality of study participants. According to WVS standards, no identifying information is collected precluding the need for written informed consent. However, to take into consideration literacy levels of the respondents, all participants also provide verbal informed consent prior to answering the survey. which is witnessed by the interviewer and documented through proceeding with the survey. As this study uses secondary datasets that have been de-identified and are openly available through the WVS website,^59^ ethical approvals from the participating countries were not necessary according to the WVS Code of Professional Ethics and Practices. Nevertheless, ethical approval for analysing data in Sweden that has been collected abroad was granted by the Swedish Ethical Review Authority (Dnr 2020-05314; decision date 2020-10-28). The study adhered to the principles embodied in the Declaration of Helsinki.

## Results

### Sociodemographic characteristics of the study population

After removing respondents with missing values (Figure 1), a total of 3,096 respondents were retained in the sample (Ethiopia *n* = 943, Kenya *n* = 1010, Zimbabwe *n* = 1143). Table 1 outlines the distribution of high (53%) vs. low reproductive agency (47%) by sociodemographic characteristics of the respondents. Close to two-thirds (64%) of respondents from Zimbabwe and around half (53%) of the Ethiopian respondents rated their reproductive agency as high, compared to only 42% of the Kenyan respondents. Respondents with higher levels of education and higher subjective social class reported slightly higher reproductive agency. Only among Muslim respondents did a minority (43%) report high reproductive agency, compared to Christians and other or no religions where 55% and 62% of respondents reported high reproductive agency respectively. Only the respondent’s country, level of education, subjective social class, and religion were statistically significantly associated with their level of reproductive agency (Table 1).

**Table 1.** Sample characteristics by levels of subjective reproductive agency (RA) in Ethiopia, Kenya, and Zimbabwe (*N* = 3096)

Characteristic	Low RA *n* = 1441 (47%)	High RA *n* = 1655 (53%)	*p*-value (chi^2^)	Total *n* = 3096 (100%)
	*n* (%)	*n* (%)		*n* (%)
**Country**			<0.001	
Ethiopia	444 (47%)	499 (53%)		943 (100%)
Kenya	584 (58%)	426 (42%)		1010 (100%)
Zimbabwe	413 (36%)	730 (64%)		1143 (100%)
**Total**	**1441 (47%)**	**1655 (53%)**	** **	**3096 (100%)**
*Missing* N (%)	*0*	*0*	* *	*0*
**Sex**	** **	** **	0.101	
Female	726 (48%)	785 (52%)	** **	1511 (100%)
Male	715 (45%)	870 (55%)		1585 (100%)
**Total**	**1441 (47%)**	**1655 (53%)**	** **	**3096 (100%)**
*Missing* N (%)	*0*	*0*	* *	*0 (100%)*
**Age**	* *	* *	0.830	* *
18–24 years	407 (48%)	440 (52%)	* *	847 (100%)
25–29 years	273 (45%)	335 (55%)	* *	608 (100%)
30–39 years	360 (46%)	416 (54%)	* *	776 (100%)
40–49 years	196 (46%)	229 (54%)	* *	425 (100%)
50–99 years	200 (46%)	232 (54%)	* *	432 (100%)
**Total**	**1436 (47%)**	**1652 (53%)**	* *	**3088 (100%)**
*Missing* N (%)	*5* (*63%)*	*3* (*37%)*	* *	*8 (100%)*
**Residence**			0.650	
Urban	551 (46%)	646 (54%)		1197 (100%)
Rural	890 (47%)	1009 (53%)		1899 (100%)
**Total**	**1441 (47%)**	**1655 (53%)**	** **	**3096 (100%)**
*Missing* (%)	*0*	*0*	*** ***	*0 (100%)*
**Educational level**			0.012	
Primary or lower	738 (49%)	764 (51%)		1502 (100%)
Secondary	484 (45%)	596 (55%)		1080 (100%)
Tertiary	215 (42%)	292 (58%)		507 (100%)
**Total**	**1437 (47%)**	**1652 (53%)**	** **	**3089 (100%)**
*Missing N (%)*	*4* (*57%)*	*3* (*43%)*	*** ***	*7 (100%)*
**Relationship status**	* *	* *	0.090	* *
Married or cohabiting	836 (45%)	1011 (55%)	*** ***	1847 (100%)
Divorced, separated, or widowed	140 (45%)	168 (55%)	*** ***	308 (100%)
Single	464 (50%)	472 (50%)	*** ***	936 (100%)
**Total**	**1440 (47%)**	**1651 (53%)**	*** ***	**3091 (100%)**
*Missing* N (%)	*1* (*20%)*	*4* (*80%)*	*** ***	*5 (100%)*
**Subjective social class**			0.044	
Upper & upper middle-class	269 (42%)	367 (58%)		636 (100%)
Lower-middle class	506 (48%)	551 (52%)		1057 (100%)
Lower & working class	654 (48%)	713 (52%)		1367 (100%)
**Total**	**1429 (47%)**	**1631 (53%)**	** **	**3060 (100%)**
*Missing* N (%)	*12* (*33%)*	*24* (*67%)*	* *	*36 (100%)*
**Religion**			<0.001	
Christian	1038 (45%)	1271 (55%)		2309 (100%)
Muslim	316 (57%)	242 (43%)		558 (100%)
No/Other religions	87 (38%)	140 (62%)		227 (100%)
**Total**	**1441 (47%)**	**1653 (53%)**	** **	**3094 (100%)**
*Missing* N (%)	*0*	*2* (100%)	* *	*2 (100%)*
**Have number of children**			0.909	
No children	468 (47%)	535 (53%)		1,003 (100%)
1 child	233 (45%)	282 (55%)		515 (100%)
2–3 children	422 (47%)	472 (53%)		894 (100%)
4 and more	314 (46%)	366 (54%)		680 (100%)
**Total**	**1437 (46%)**	**1655 (54%)**	** **	**3092 (100%)**
*Missing* N (%)	*4* (100%)	*0*	* *	*4* (100%)

### Descriptive statistics of reproductive agency and values and attitudes toward SRHR and gender equality

The distribution of attitudes toward SRHR among male and female respondents across the three countries has been reported in previous studies,^63^ and therefore will only briefly be repeated here. As mentioned, the SRHR Support Index is measured on a scale from 0 to 100, where lower numbers indicate low support for SRHR. Overall, there was low support for SRHR with a mean value at 39.2 (SD = 15.3).^63^ The differences between respondents were significant for country, but not for sex. Respondents in Kenya had the highest score, followed by Ethiopia, and the lowest scores were found in Zimbabwe. For the Equality Index, respondents from Ethiopia scored higher than those from other countries, as did women compared to male respondents (Appendix Table 1A).

Table 2 shows the distribution of attitudes toward SRHR and gender equality for respondents who reported low vs. high reproductive agency (also visualised in a panel of histograms, Appendix Figure 3A). The mean support among both respondents with low reproductive agency (mean 39.7, SD 15.2) and high reproductive agency (38.7, SD 16.1) was similar to that of the full sample (mean 39.2, SD 15.3). Mean values for the sub-indices and the WVS Equality Index was higher on average than for SRHR in general, except in the case of Sexual and Reproductive Rights (Table 2). This dimension of SRHR received the lowest support on average (mean 15.7, SD 23.45), with slightly higher support among those expressing low (16.9, SD 22.3) than high reproductive agency (mean 14.7, SD 24.3). Similarly, the support for Neighborhood Sexual Safety was higher among those with low (mean 60.4, SD 28.3) than those with high (mean 54.6, SD 28.9) reproductive agency. Equitable Masculinity had the highest support (mean 66.0, SD 16.9), more so among those with high (mean 68.2, SD 16.1) than low (mean 63.4, SD 17.5) reproductive agency. Respondents who were more supportive of SRHR interventions, and gender equality as measured by the WVS Equality Index, also tended to report higher reproductive agency. However, when using the Gender Equitable Relationship SRHR sub-index, support for gender equality did not vary by reproductive agency (mean 48.1, SD 18.4 among those with low agency; mean 47.9, SD 18.0 among those with high agency). These patterns suggest heterogeneity in the crude relationship between individuals’ subjective reproductive agency and the values and attitudes toward distinct dimensions of SRHR and gender equality.

**Table 2.** Distribution of support for SRHR and gender equality values and attitudes by levels of subjective reproductive agency (RA) in Ethiopia, Kenya, and Zimbabwe (*N* = 3096)

Variables	Summary Statistics	Low RA *n* = 1441	High RA *n* = 1655	Total *n* = 3096
SRHR Support Index	Mean (SD) Median (IQR)	39.75 (15.23) 37.06 (29.77–46.75)	38.68 (16.12) 35.25 (28.04–45.47)	39.18 (15.72) 36.20 (28.96–46.04)
Sexual and Reproductive Rights sub-index	Mean (SD) Median (IQR)	16.89 (22.38) 6.45 (0.93–26.20)	14.68 (24.31) 3.12 (0.86–16.90)	15.71 (23.45) 4.68 (0.89–20.69)
Neighborhood Sexual Safety Norm sub-index	Mean (SD) Median (IQR)	60.43 (28.29) 63.85 (39.76–86.84)	54.60 (28.90) 57.43 (33.56–77.84)	57.32 (28.76) 59.44 (35.91–81.42)
Gender Equitable Relationships sub-index	Mean (SD) Median (IQR)	48.15 (18.38) 47.92 (35.61–60.86)	47.95 (18.04) 45.97 (35.88–59.62)	48.05 (18.20) 47.07 (35.75–60.26)
Equitable Masculinity Norms sub-index	Mean (SD) Median (IQR)	63.38 (17.53) 61.86 (54.63–78.95)	68.22 (16.06) 68.74 (57.66–82.50)	65.97 (16.93) 64.81 (56.36–81.01)
SRHR Interventions sub-index	Mean (SD) Median (IQR)	48.51 (15.39) 47.65 (38.51–58.56)	51.78 (15.06) 50.15 (41.81–61.86)	50.26 (15.30) 48.85 (40.36–60)
Equality index	Mean (SD) Median (IQR)	58.20 (23.45) 58.33 (44–72)	60.45 (23.13) 63.66 (44–77.33)	59.41 (23.30) 60.66 (44–75)

### Values and attitudes toward SRHR and their association with reproductive agency

We then proceeded with logistic regression analysis to explore whether values and attitudes toward SRHR and gender equality predict respondents’ reproductive agency (Table 3). In bivariate analyses, support for Sexual and Reproductive Rights and Neighborhood Sexual Safety were associated with lower odds of high reproductive agency, while support for Equitable Masculinity Norms, SRHR Interventions, and Gender Equality were associated with higher odds. Likely due to the opposite directions of these coefficients, the association with SRHR overall was not significant, nor with Gender Equitable Norms.

**Table 3.** Bivariate and adjusted logistic regressions of the association between supportive values and attitudes toward SRHR, gender equality and reproductive agency for the total study population

Variables	cOR (95% CI)	aOR (95% CI)
Support for SRHR Support Index	0.99 (0.99–1.00)	1.004 (0.999–1.010)
Support for SRR sub-index	0.99** (0.99–1.00)	1.002 (0.998–1.005)
Support for Neighborhood Sexual Safety sub-index	0.99*** (0.99–0.99)	0.993*** (0.991–0.996)
Support toward Gender-Equitable Relationships sub-index	0.99 (0.99–1.00)	1.003 (0.998–1.007)
Support for Equitable Masculinity Norms sub-index	1.02*** (1.01–1.02)	1.01*** (1.01–1.02)
Support for SRHR Interventions sub-index	1.01*** (1.00–1.02)	1.02*** (1.01–1.02)
Support for Gender Equality Index	1.004** (1.001–1.007)	1.004* (1.0003–1.01)

*p*-value * < 0.05, ** < 0.01, *** < 0.001*.*

Note: Covariates include age, sex, residency, education, relationship status, religion, subjective social class, number of children, and country.

Most of these relationships held when adjusting for sociodemographic variables, except for attitudes to Sexual and Reproductive Rights that were no longer significantly linked to reproductive agency. The associations between reproductive agency and support for Equitable Masculinity Norms (aOR 1.01, 95% CI 1.01–1.02), SRHR Interventions (aOR 1.02, 95% CI 1.01–1.02), as well as Gender Equality (aOR 1.004, 95% CI 1.0003–1.01) were statistically significant. In substantive terms, this means that for each increase of 1 in support for gender equality and universal access to SRHR services, the odds of reporting high reproductive agency are 0.4–2% higher. Considering that these are 100-graded scales, these are not negligible coefficients.

In contrast, perceiving one’s neighbourhood as safe from men’s violence and harassment of women and girls was associated with 0.7% lower odds of reporting high reproductive agency for each increase of 1 in support on the 100-graded scale (aOR 0.993, 95% CI 0.991–0.996). Again, the relationship with attitudes to SRHR overall was not significant, likely due to heterogeneity by the dimension of SRHR.

In robustness checks, linear probability models mirrored these results except for attitudes to overall SRHR and Gender-Equitable Relationships, which were both significantly associated with higher reproductive agency (both by 0.8 percentage points, 95% CI 0.002–0.014) (Appendix Table 2A).

We also tested a logistic model adding fixed effects for regions to address local norms and characteristics (Table 3A). The associations with attitudes to Equitable Masculinity, SRHR Intervention, and Neighborhood Sexual Safety were mostly the same as in the main model (Table 3). However, the association with Gender Equality was no longer significant, while overall SRHR was, albeit with a negligible odds ratio (aOR 1.008, 95% CI 1.002–1.014). This suggests that the relationship between values and attitudes to Gender Equality and perceived reproductive agency is sensitive to local contextual factors, like social norms.

When we stratified the adjusted logistic regression models by country and sex (Table 4), findings suggest that support for SRHR overall (aOR 1.03, 95% CI 1.02–1.04) and Sexual and Reproductive Rights (aOR 1.02, 95% CI 1.01–1.02) was positively associated with the odds of reporting high reproductive agency in Ethiopia. In Kenya, the relationship with Sexual and Reproductive Rights was negative (aOR .99, 95% CI .98–.99). Neighborhood Sexual Safety was associated with lower odds of reporting high reproductive agency in Ethiopia (aOR .99, 95% CI .98–.99) and Zimbabwe (aOR .99, 95% CI .99–.99), as well as among both male (aOR .99, 95% CI .99–.99) and female participants (aOR .99, 95% CI .99–.99). This was the only statistically significant relationship observed in Zimbabwe. Respondents in Ethiopia and Kenya who expressed more support for Equitable Masculinity (aOR 1.02, 95% CI 1.01–1.03 in Ethiopia; aOR 1.03, 95% 1.02–1.04 in Kenya) and SRHR Interventions (aOR 1.02, 95% CI 1.01–1.03 in Ethiopia; and aOR 1.02, 95% CI 1.01–1.02 in Kenya) had higher odds of reporting high reproductive agency. In all but one dimension of attitudes to SRHR and gender, there were similar results between the sexes; only among male respondents did supportive attitudes for Gender-Equitable Relationships correlate with high reproductive agency (aOR 1.01, 95% CI 1.00–1.01).

**Table 4.** Adjusted logistic regressions of the association between supportive values and attitudes toward SRHR, gender equality and reproductive agency, stratified by country and sex of respondents

Support for SRHR	Ethiopia aOR (95% CI)	Kenya aOR (95% CI)	Zimbabwe aOR (95% CI)	Male aOR (95% CI)	Female aOR (95% CI)
SRHR Support Index	1.03*** (1.02–1.04)	1.00 (.988–1.00)	1.00 (.99–1.00)	1.00 (.997–1.012)	1.00 (.998–1.013)
Sexual and Reproductive Rights sub-index	1.02*** (1.010–1.024)	.99*** (.983–.995)	1.00 (.995–1.007)	1.00 (.996–1.006)	1.00 (.998–1.007)
Neighborhood Sexual Safety sub-index	.99*** (.982–.994)	1.00 (.99–1.00)	.99* (.989–.999)	.99** (.990–.998)	.99** (.990–.998)
Gender Equitable Relationships sub-index	1.01 (.999–1.014)	1.00 (.995–1.011)	1.00 (.99–1.00)	1.01* (1.000–1.013)	1.00 (.993–1.006)
Equitable Masculinity Norms sub-index	1.02*** (1.010–1.027)	1.03*** (1.020–1.036)	1.00 (.99–1.00)	1.01*** (1.005–1.018)	1.02*** (1.011–1.025)
SRHR Interventions sub-index	1.02*** (1.013–1.033)	1.02*** (1.009–1.024)	1.01 (.996–1.017)	1.02*** (1.012–1.027)	1.01*** (1.005–1.020)
WVS Equality Index	1.01** (1.00–1.014)	1.01* (1.00–1.01)	0.99 (.99–1.00)	1.00 (.99–1.01)	1.00 (.99–1.01)

*p*-value * < 0.05, ** < 0.01, *** < 0.001*.*

Note: Covariates include age, sex, residency, education, relationship status, religion, subjective social class, number of children. In models stratified by sex, country was also included. In models stratified by country, sex was also included.

## Discussion

### Summary of findings

This study assessed the relationship between individual-level values and attitudes toward SRHR and gender equality with the level of perceived reproductive agency among women and men in Ethiopia, Kenya, and Zimbabwe.

We found that people generally reported high perceived reproductive agency, mirroring previous research.^10^ In line with our hypothesis, the findings point to the relevance of values and attitudes toward SRHR and gender equality for people’s reproductive agency, regardless of socioeconomic factors.^12,16,38,52,66,67^ Respondents who were more supportive of Gender Equality, Equitable Masculinity, and SRHR Intervention norms were more likely to report high reproductive agency, even when adjusting for possible confounders. In contrast, respondents who perceived their neighbourhoods as safe from GBV and sexual harassment reported lower reproductive agency.

### Sociodemographic factors and reproductive agency

We found that people generally reported high perceived reproductive agency, mirroring previous research finding that about 90% of women in Ethiopia, Kenya, and Zimbabwe have the power to decide over their contraceptive use.^10^ Contradictory to what may be expected, neither sex nor age statistically significantly correlated with the level of reproductive agency in our study, which is interesting since most research exclusively focuses on women. While women’s ability to make choices is particularly relevant to childbearing, considering the biological nature of reproduction, this finding points perhaps to the limitations of simply equating women’s empowerment with reproductive empowerment and serves as a reminder of how complex agency in fertility is, and how caution is necessary when navigating this issue.^12^ Although sex and age were not statistically significantly associated, country, level of education, subjective social class, and religion tended to significantly predict reproductive agency. These results support previous research findings and the ICRW framework on reproductive empowerment which suggest how education and subjective social class,^12,53,58^ religion and country,^55,56^ are associated with empowerment. These four variables can be seen as some of the key individual and structural factors shaping a person’s opportunities in life.^12,13,44^ Access to resources and capacity-enhancing experiences (captured by social class and education) may influence a person’s decision-making ability, while the material, socio-political, and cultural contexts – represented by country and religion – impact norms and power distributions in society that enable or hinder the ability of individuals to make use of those resources and capacities.^13^

### Heterogeneity in findings

Country-level differences were more prominent than sex differences, which points to the importance of normative contexts. For example, supportive values and attitudes toward a range of SRHR dimensions were more often associated with a high sense of reproductive agency in Ethiopia, to a lesser extent in Kenya, and almost not at all in Zimbabwe. Most consistent was the relationship between supporting Equitable Masculinity norms and SRHR Intervention norms and reporting high reproductive agency, regardless of sex and country (except Zimbabwe).

In Kenya, support for sexual and reproductive rights was associated with lower reproductive agency. People who hold more supportive attitudes toward sexual and reproductive rights may be more likely to critically view their opportunity to exercise such rights.^51,68–70^ As such, they may perceive their reproductive agency as limited.

The only sex difference we observed was a positive association between supporting Gender Equitable Relationships and reporting high reproductive agency among men only, not women. This adds to the literature on how men’s values and attitudes are important for SRH outcomes and their own well-being.^39,44^ Previous studies have shown that harmful masculinity attitudes are associated with depression and suicidality among young men in the UK, the US, and Mexico,^71^ while equitable masculinity attitudes are associated with higher life satisfaction and self-esteem among men in Jordan,^72^ the UK, and the US.^71^

We captured values and attitudes to SRHR interventions including comprehensive sexuality education, contraceptive availability also for unmarried people, safe abortion in case of an unwanted pregnancy, and infertility treatment. Our findings suggest that supportive values and attitudes to such interventions correlate with a sense of high reproductive agency among both men and women, echoing previous studies in Kenya,^73^ Ethiopia,^74,75^ Uganda,^76^ and the US.^77^ Our study expands this literature by showing that values and attitudes play a role in shaping not only *a priori*-defined measures of agency, but also people’s own understanding of their realities and abilities to fulfil their SRHR.

While the results generally supported our hypothesis, there were some unexpected and contradictory findings. Respondents who perceived their neighbourhoods as safe from gender-based violence and sexual harassment had lower reproductive agency. While our theoretical framework cannot explain this association, results indicate that neighbourhood safety is not directly related to people’s control of their reproductive lives, pointing to the complex and dynamic process between social norms, individual values, and reproductive agency. Since we did not consider aspects of personal safety, more research is needed to explore how violence in other settings – such as the home or workplace – may impact one’s perception of reproductive agency.^12,45^

### Strengths and limitations

This study is based on a collaboration with a well-established global survey program on norms and values. The main strengths of this data source include the nationally representative sampling of both men and women regardless of marital status or sexual orientation, high validity and reliability, and high response rates. However, because the WVS collects data through face-to-face interviews, there is a risk of social desirability as participants may provide answers that are most accepted in their setting. Still, the use of experienced interviewers who have had thorough training by the WVS may limit this bias. Moreover, the use of cross-sectional data also precludes any causal interpretations of the relationship between attitudes toward SRHR and high reproductive agency.

Although our results were small, the 100-graded scale of our independent variables (attitudes) meant that even a small odds ratio is meaningful. The R-squares show that a small portion of variance is explained by the independent variables and covariates. Despite likely missing several factors influencing reproductive agency, our results align with previous research as discussed earlier.

This study focused on people’s own assessments of their freedom of choice and control over their reproductive lives, without judgment of what this may mean for that person, providing meaningful cross-sectional information to understand reproductive agency as a subjective experience with intrinsic value. While this self-reported indicator is novel and useful, it also has limitations. Reproductive agency is a comprehensive concept that may shift over time. The item we used does not capture the meaning that people attribute to this concept and the implications this may have for their actions, achievement of goals, or the impact on their health.^12^ As such, it does not reflect how “free choice” can mean different things to different people in different situations. Dichotomising this item using the total sample median obscures the slight variations observed across countries and sexes. The consistently high rating by women may hide inequalities highlighted by much previous research on reproductive empowerment and agency. More research is needed to assess the concordance between self-reported and externally defined measures of reproductive agency (and empowerment).

Measuring attitudes and their health implications is challenging. Not only due to social desirability bias, but also because of the discrepancies between “abstract” attitudes and behaviour in practice.^78, 79^ People do not always act according to their values, due to social pressure and structures to ensure compliance with social norms.^78^ This may have implications for measuring values and attitudes in quantitative research. The partially contradictory findings from this study could indicate potential gaps between attitudes and practice.^79^ It is also possible that social desirability bias makes people report what they perceive are appropriate answers (social norms) rather than what they believe themselves (personal values and attitudes). Future research may explore whether attitudes of other people living in the nearby surroundings (i.e. local norms) are more salient for reproductive agency, since we might expect that people’s ability to make decisions related to reproduction is affected by opportunity structures in the area where they live.

We note that in these three very different study countries, the median value for reproductive agency was consistently 8 or higher on a scale from 1 to 10. This could indicate limitations of applying terminology developed in the minority world/Global North to other contexts; theories and concepts related to gender may not be universally applicable.^47–50,80^ For example, there have been critiques of an overemphasis on hegemonic masculinity in interventions in many parts of Africa at the expense of exploring and understanding other ways of conceptualising masculinity.^48–50^

Despite its limitations, our study contributes to the literature as the first study – to our knowledge – to examine reproductive agency through a single self-reported item in different sub-Saharan countries. Our study adds new knowledge about how people – regardless of marital status, sex, or age – perceive their own sense of reproductive agency and how it is associated with their values and attitudes toward a wide range of SRHR dimensions and gender equality. The item is now integrated into the next round of the WVS, facilitating systematic global data collection and comparison, a challenge faced by other scales using individual measures of reproductive agency.^32^ The study thus contributes to research about reproductive empowerment, including additional items to measure and understand it.^12,16,67,81–84^

While there is little prior research on perceived reproductive agency, vast research indicates that subjective assessments of well-being are associated with longer, healthier, and more productive lives and supportive relationships.^85^ As such, a better understanding of reproductive agency could prove useful to policies, practices, research, and interventions to ensure SRHR for all. With the addition of this item to the next wave of the WVS, further in-depth analyses are made possible, including exploring possible implications for people's access to and fulfilment of SRHR and gender equality. This may be particularly relevant as research has indicated unclear associations between (reproductive) empowerment and the use of modern birth control or having one’s ideal number of children.^12,53^ We also see research opportunities for combining WVS with other datasets – for example, the Demographic Health Surveys^56^ – to carry out socioecological studies that can speak to aggregate patterns in behaviour, values and attitudes.

## Conclusion

Our results suggest that values and attitudes toward SRHR and gender equality are linked to individuals’ perceived freedom of choice and control over their reproductive life. The relationship between attitudes and reproductive agency is complex, as supportive views can sometimes be associated with lower reproductive agency. Given the intrinsic link between sexual and reproductive health and rights, values and attitudes towards such rights must be explicitly and constructively addressed in interventions aiming at fulfilling SRHR for all. Yet, the variations observed across countries, underscore the need for a nuanced understanding and context-specific considerations when addressing the intricate relationships between values, attitudes, gender equality, and reproductive agency in diverse sociocultural settings.

We echo previous recommendations to the family planning and SRHR community, urging it to review and implement cross-cultural measures to monitor progress.^28,35^ It is important to critically reflect on the extent to which these measures are aligned with the underlying goals of SRHR as defined by the Maputo Protocol, Agenda 2030, and the Guttmacher-Lancet definition of SRHR, and whether these can be complemented with self-assessment items out of respect for the respondents’ integrity and the intrinsic value of reproductive agency.

On a final note, we encourage readers to see this analysis as a step toward global measurements of values and attitudes toward SRHR and gender equality and reproductive agency as many of these items are now included in the new core WVS questionnaire, which is currently being implemented across the globe. Such research could explore how it can be useful in understanding subjective assessments of agency (through other or no cut-off points), given the free availability of the data on the WVS website.

## Supplementary Material

Supplemental Figures A1-3

Supplemental Tables A1-3

## Figures and Tables

**Figure 1A.**
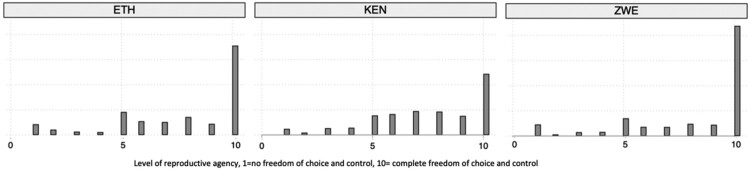
Distribution of reproductive agency by country, 1 = No freedom of choice and control of family planning, 10 = Complete freedom of choice and control of family planning

**Figure 2A.**
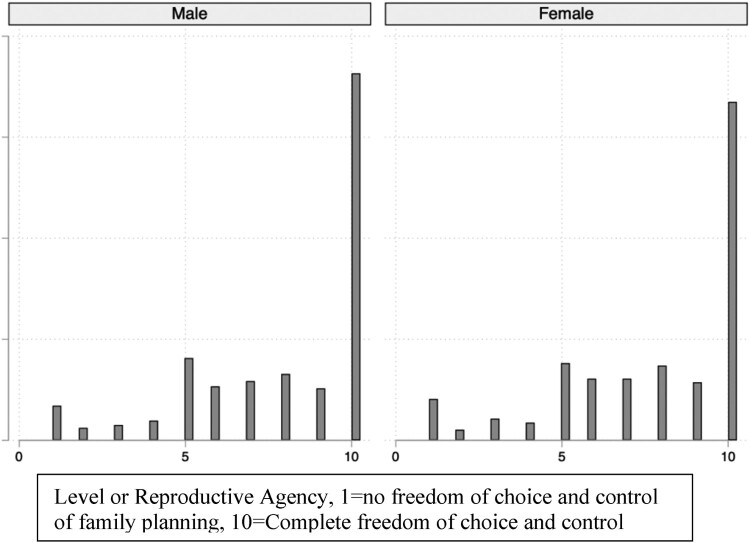
Distribution of reproductive agency by sex, 1 = No freedom of choice and control of family planning, 10 = Complete freedom of choice and ontrol of family planning

**Figure 3A.**
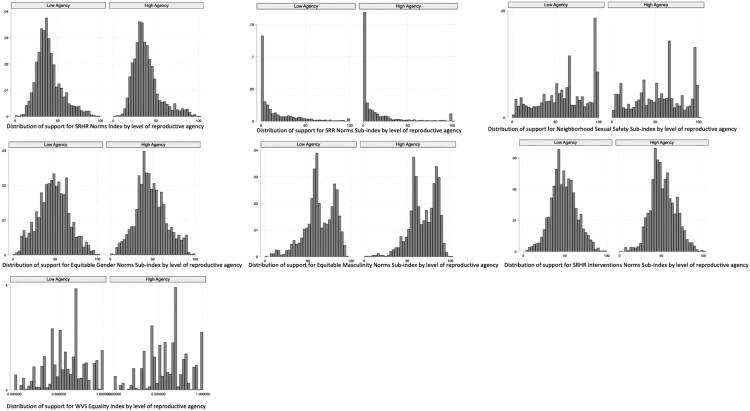
Panel of histograms showing the distribution of support for the SRHR Support Index, its five subindices, and the WVS Equality Index, by level of reproductive agency

## Footnotes

1 We did not include measures of employment or family savings due to multicollinearity with subjective social class.

## Provenance statement

This paper was not commissioned and went through external peer review.

## Appendix

**Table 1A.** Attitudes toward gender equality as measured by the WVS Equality Index (score from 0–100, where 0 = no support for gender equality and 100 = full support for gender equality), by country and sex of respondent

Variable	Mean (SD)	Median (IQR)
Ethiopia	61.48 (24.16)	66.66 (44.33–77.33)
Kenya	59.47 (23.63)	61 (44–77.33)
Zimbabwe	57.64 (22.14)	58.33 (41.33–69.33)
Men	55.94 (23.72)	58 (41.33–69)
Women	63.04 (22.30)	66.66 (49.66–77.66)
Total	59.41 (23.30)	60.66 (44–75)

**Table 2A.** Bivariate and adjusted linear regression models of the association between supportive values and attitudes toward SRHR and gender equality and reproductive agency for the total study population

Variables	Model 1 B (95% CI)	Model 2 B (95% CI)
Support for SRHR Support Index	.001 (−.005–.007)	.008* (.002–.014)
R-squared	0.000	0.073
Constant	7.76	8.14
Support for SRR sub-index	−.004 (−.008–.0002)	.002 (−.002–.006)
R-squared	0.001	0.071
Constant	7.85	8.44
Support for Neighbourhood Sexual Safety sub-index	−.009*** (−.012–−.005)	−.007*** (−.010–−.003)
R-squared	0.009	0.075
Constant	8.29	8.95
Support toward Gender-Equitable Relationships sub-index	.009*** (.004–.015)	.008** (.002–.014)
R-squared	0.004	0.074
Constant	7.34	8.08
Support for Equitable Masculinity Norms sub-index	.025*** (.019–.030)	.019*** (.014–.025)
R-squared	0.025	0.085
Constant	6.14	7.27
Support for SRHR Interventions sub-index	.025*** (.019–.031)	.022*** (.015–.023)
R-squared	0.021	0.085
Constant	6.52	7.25
Support for Equality Index	.009*** (.005–.013)	.007** (.003–.011)
R-squared	0.006	0.074
Constant	7.26	8.09

* *p* < 0.05, ** *p* < 0.01, *** *p* < 0.001, B = coefficient, CI = confidence intervals.

Note: Table XXA shows results from bivariate (Model 1) and adjusted (Model 2) linear probability regression models of supportive attitudes toward SRHR in relation to reproductive agency. Covariates included in Model 2 are the respondent’s age, sex, place of residency, education, relationship status, religion, subjective social class, number of children, and country.

**Table 3A.** Adjusted odds ratios from fixed effects logistic regression analysis of the association between supportive values and attitudes toward SRHR, gender equality and reproductive agency controlling for within country region

Variables	aOR (95% CI)
Support for SRHR Support Index	1.008** (1.002–1.014)
Support for SRR sub-index	1.005** (1.001–1.009)
Support for Neighbourhood Sexual Safety sub-index	.993*** (.990–.997)
Support toward Gender-Equitable Relationships sub-index	1.005 (.999–1.01)
Support for Equitable Masculinity Norms sub-index	1.01*** (1.006–1.016)
Support for SRHR Interventions sub-index	1.014*** (1.008–1.020)
Support for Gender Equality Index	1.20 (.85–1.71)

* *p* < 0.05, ** *p* < 0.01, *** *p* < 0.001, aOR = Adjusted Odds Ratio, CI = confidence intervals.

Note: Covariates included the respondent’s age, sex, residency, education, relationship status, religion, subjective social class, number of children, but not country. In total, 2 groups (23 observations) were omitted due to all positive or all negative outcomes.
