# Supplementary material for: Attitudes toward sexual and reproductive health and rights and their associations with reproductive agency: a population-based cross-sectional study in Ethiopia, Kenya, and Zimbabwe
Source: Sex Reprod Health Matters. 2025 Jan 13;32(1):2444725. doi: 10.1080/26410397.2024.2444725 (PMC11849024; doi:10.1080/26410397.2024.2444725)
Supplement: Supplemental Figures A1-3 [file ZRHM_A_2444725_SM8236.docx]

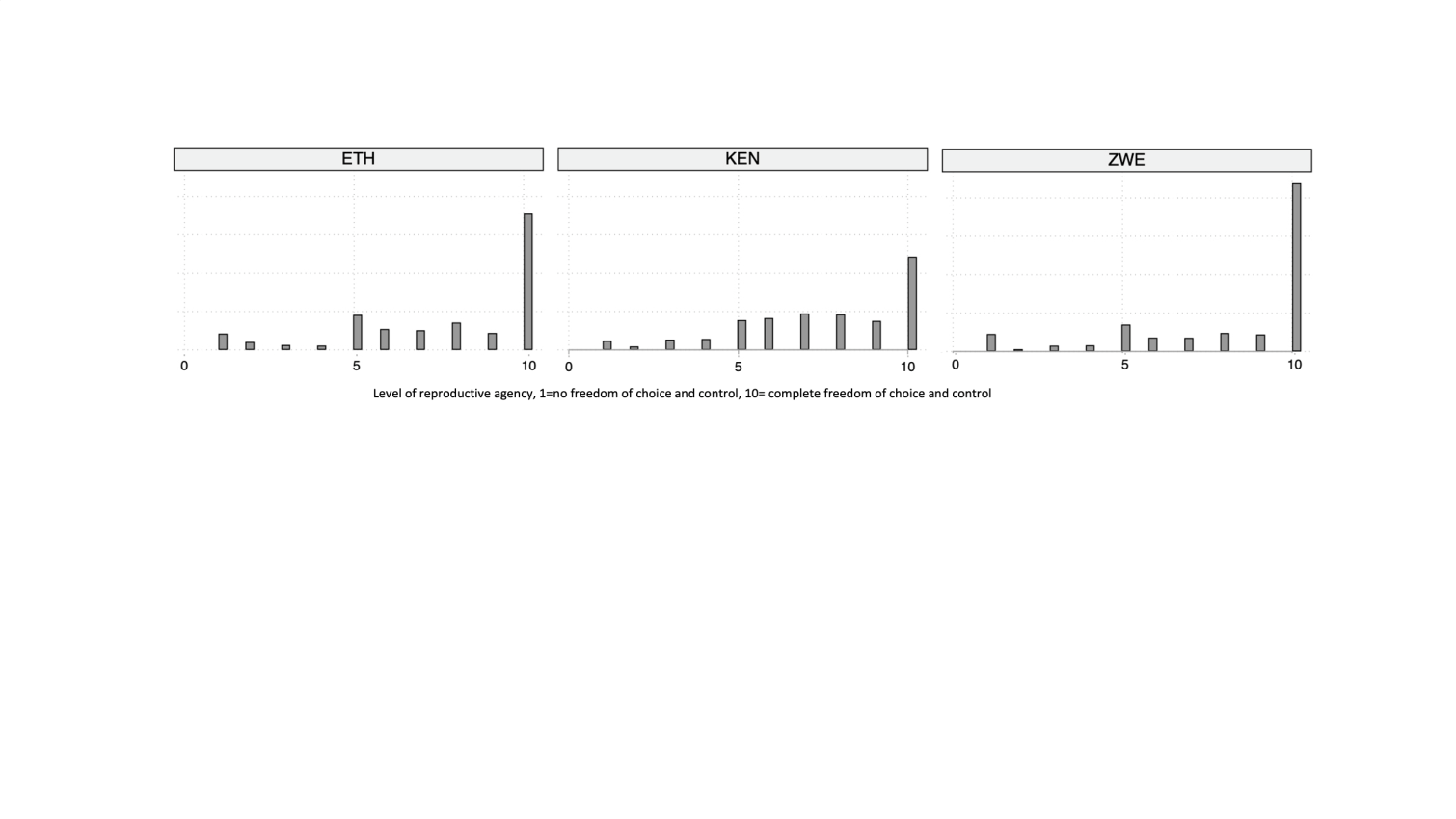


**Figure 1A: Distribution of reproductive agency by country, 1 = No freedom of choice and control of family planning, 10 = Complete freedom of choice and control of family planning**

***
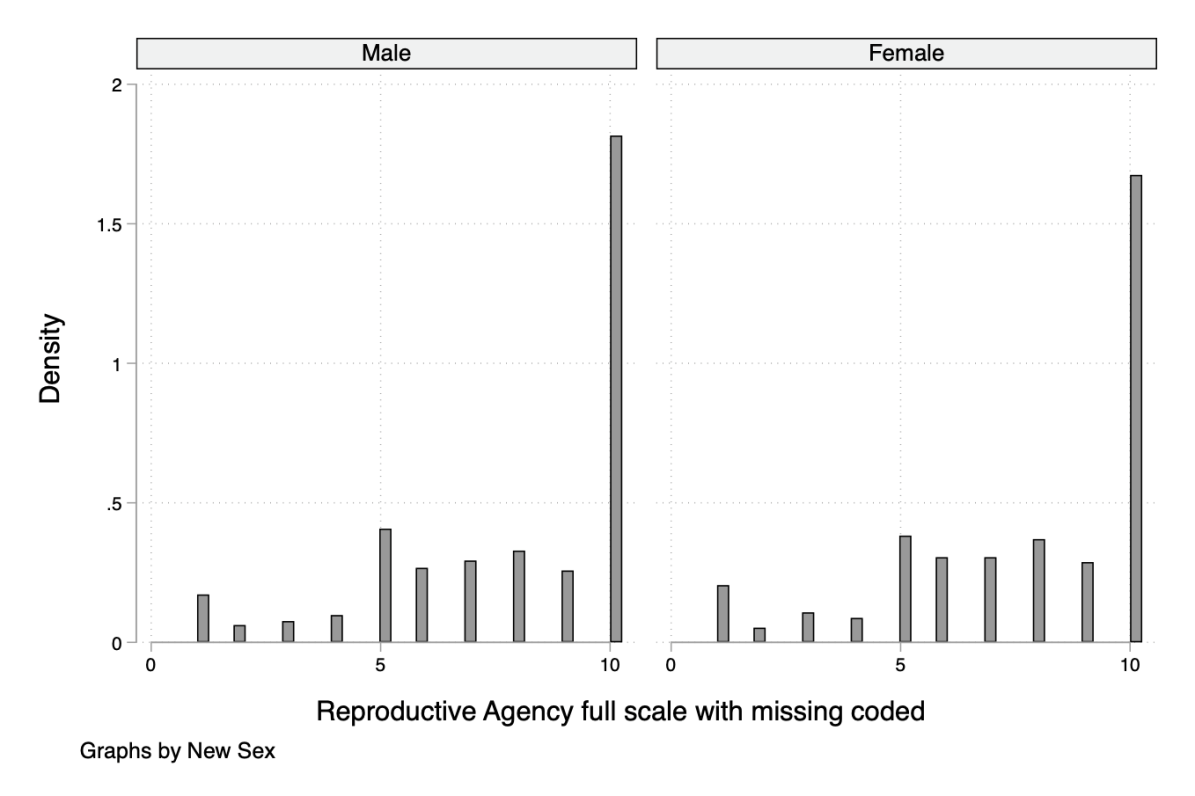
***

Level or Reproductive Agency, 1=no freedom of choice and control of family planning, 10=Complete freedom of choice and control

**Figure 2A: Distribution of reproductive agency by sex, 1 = No freedom of choice and control of family planning, 10 = Complete freedom of choice and control of family planning**

**
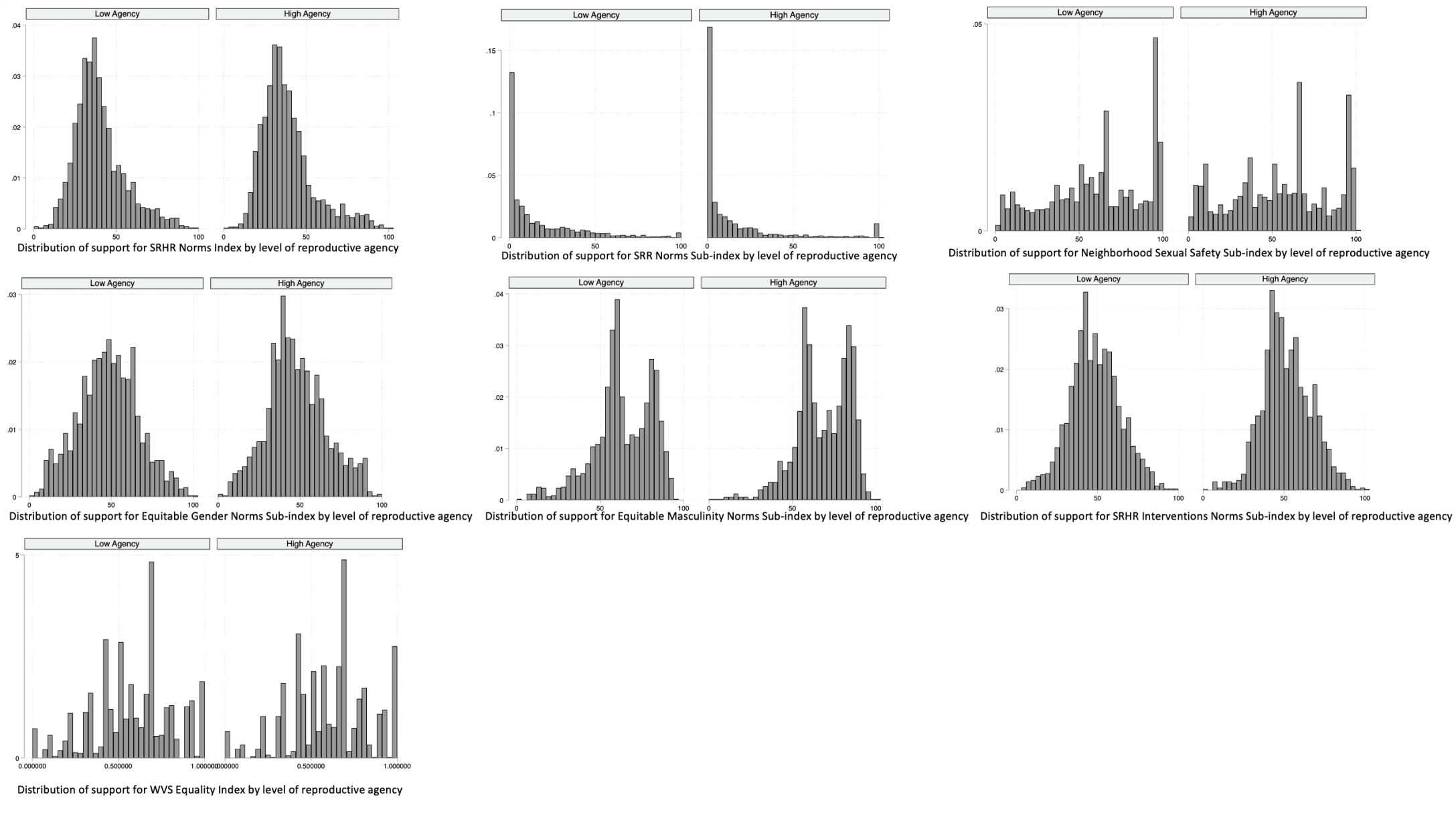
Figure 3A: Panel of histograms showing the distribution of support for the SRHR Support Index, its five subindices, and the WVS Equality Index, by level of reproductive agency.**
