# Supplementary material for: Attitudes toward sexual and reproductive health and rights and their associations with reproductive agency: a population-based cross-sectional study in Ethiopia, Kenya, and Zimbabwe
Source: Sex Reprod Health Matters. 2025 Jan 13;32(1):2444725. doi: 10.1080/26410397.2024.2444725 (PMC11849024; doi:10.1080/26410397.2024.2444725)
Supplement: Supplemental Tables A1-3 [file ZRHM_A_2444725_SM8235.docx]

**Appendix**

| **Table 1A: Attitudes toward gender equality as measured by the WVS Equality Index (score from 0–100, where 0=no support for gender equality and 100=full support for gender equality), by country and sex of respondent** | | |
| --- | --- | --- |
| **Variable** | **Mean (SD)** | **Median (IQR)** |
| Ethiopia | 61.48 (24.16) | 66.66 (33) |
| Kenya | 59.47 (24.62) | 61 (33.33) |
|  |  |  |
| Zimbabwe | 57.64 (22.14) | 58.33 (28) |
| Men | 55.94 (23.72) | 58 (27.66) |
| Women | 63.04 (22.30) | 66.66 (28) |
| Total | 59.40 (23.30) | 60.66 (31) |

| \| **Table 2A: Bivariate and adjusted linear regression models of the association between supportive values and attitudes toward SRHR and gender equality and reproductive agency for the total study population** \| \| \| \| --- \| --- \| --- \| \| **Variables** \| **Model 1**  **B**  **(95% CI)** \| **Model 2**  **B**  **(95% CI)** \| \| Support for SRHR Support Index \| .001  (-.005 – .007) \| .008*  (.002 – .014) \| \| R-squared \| 0.000 \| 0.073 \| \| Constant \| 7.76 \| 8.14 \| \| Support for SRR sub-index \| -.004  (-.008 – .0002) \| .002  (-.002 – .006) \| \| R-squared \| 0.001 \| 0.071 \| \| Constant \| 7.85 \| 8.44 \| \| Support for Neighbourhood Sexual Safety sub-index \| -.009***  (-.012 – -.005) \| -.007***  (-.010 – -.003) \| \| R-squared \| 0.009 \| 0.075 \| \| Constant \| 8.29 \| 8.95 \| \| Support toward Gender-Equitable Relationships sub-index \| .009***  (.004 – .015) \| .008**  (.002 – .014) \| \| R-squared \| 0.004 \| 0.074 \| \| Constant \| 7.34 \| 8.08 \| \| Support for Equitable Masculinity Norms sub-index \| .025***  (.019 – .030) \| .019***  (.014 – .025) \| \| R-squared \| 0.025 \| 0.085 \| \| Constant \| 6.14 \| 7.27 \| \| Support for SRHR Interventions sub-index \| .025***  (.019 – .031) \| .022***  (.015 – .023) \| \| R-squared \| 0.021 \| 0.085 \| \| Constant \| 6.52 \| 7.25 \| \| Support for Equality Index \| .009***  (.005 – .013) \| .007**  (.003 – .011) \| \| R-squared \| 0.006 \| 0.074 \| \| Constant \| 7.26 \| 8.09 \| \| * p<0.05, ** p<0.01, *** p<0.001, B=coefficient, CI=confidence intervals. Table XXA shows results from bivariate (Model 1) and adjusted (Model 2) linear probability regression models of supportive attitudes toward SRHR in relation to reproductive agency. Covariates included in Model 2 are the respondent’s age, sex, place of residency, education, relationship status, religion, subjective social class, number of children, and country. \| \| \| |
| --- | --- | --- | --- | --- | --- | --- | --- | --- | --- | --- | --- | --- | --- | --- | --- | --- | --- | --- | --- | --- | --- | --- | --- | --- | --- | --- | --- | --- | --- | --- | --- | --- | --- | --- | --- | --- | --- | --- | --- | --- | --- | --- | --- | --- | --- | --- | --- | --- | --- | --- | --- | --- | --- | --- | --- | --- | --- | --- | --- | --- | --- | --- | --- | --- | --- | --- | --- | --- | --- | --- | --- | --- |

| **Table 3A: Adjusted odds ratios from fixed effects logistic regression analysis of the association between supportive values and attitudes toward SRHR, gender equality and reproductive agency controlling for within country region** | |
| --- | --- |
| **Variables** | **aOR**  **(95% CI)** |
| Support for SRHR Support Index | 1.008**  (1.002 – 1.014) |
| Support for SRR sub-index | 1.005**  (1.001 – 1.009) |
| Support for Neighbourhood Sexual Safety sub-index | .993***  (.990 – .997) |
| Support toward Gender-Equitable Relationships sub-index | 1.005  (.999 – 1.01) |
| Support for Equitable Masculinity Norms sub-index | 1.01***  (1.006 – 1.016) |
| Support for SRHR Interventions sub-index | 1.014***  (1.008 – 1.020) |
| Support for Gender Equality Index | 1.20  (.85 – 1.71) |
| * p<0.05, ** p<0.01, *** p<0.001, aOR=Adjusted Odds Ratio, CI=confidence intervals. Covariates included the respondent’s age, sex, residency, education, relationship status, religion, subjective social class, number of children, but not country. In total, 2 groups (23 observations) were omitted due to all positive or all negative outcomes. | |
